# Grammatical Gender Influences Semantic Categorization and Implicit Cognition in Polish

**DOI:** 10.3389/fpsyg.2019.02208

**Published:** 2019-10-04

**Authors:** Józef Maciuszek, Mateusz Polak, Natalia Świa̧tkowska

**Affiliations:** Institute of Applied Psychology, Faculty of Management and Social Communication, Jagiellonian University, Kraków, Poland

**Keywords:** grammatical gender, semantic categorization, triadic similarity judgments, Implicit Association Test, similarity and gender, sex and gender

## Abstract

The influence of grammatical gender on cognitive processes is an important issue in contemporary psycholinguistics and language psychology, particularly in research concerning the relations between grammar and semantics. The extent of this effect is dependent on a given language’s gender system and its grammatical specifics. The aim of the presented research was to investigate grammatical gender effects in Polish – a Slavic language with three singular and two plural grammatical genders. In Experiment 1, triadic similarity judgments were used, and it turned out that the grammatical gender of nouns influenced perceived similarity of words in case of animals, but not inanimate objects or abstract concepts. In Experiment 2 we used a modified Implicit Association Test; results suggest that grammatical gender seems to be of implicit nature, as grammatical gender consistency influenced reaction times and the number of classification errors. In Experiment 3 participants assigned male and female voices to animals and inanimate objects, which were presented either as words or as pictures. Grammatical gender effects occurred for both animate and inanimate objects and were similar for verbal and visual stimuli. It turned out that in the Polish language the influence of grammatical gender may occur on the lexicosemantic level and the conceptual level, and concerns both animate and inanimate objects. Results are discussed in context of the *similarity and gender* and the *sex and gender* hypotheses.

## Introduction

The notion that language affects cognition has a long history. This idea was popularized due to the development of cultural linguistics at the turn of the 19th century and the work of such researchers as F. Boas, E. Sapir, and B. L. Whorf, who perceived grammatical structures as the source of differences in the way of thinking, learning and experiencing reality (Whorf, 1956 – *the principle of linguistic relativity*). Nowadays, numerous studies confirm that language has an impact on cognition (Lucy, 1992), and one of the factors influencing it is grammatical gender (see Boroditsky et al., 2003; Vigliocco et al., 2005; Cubelli et al., 2011, for a review). However, apart from a strong version, which assumes that language strictly determines thinking and that language categories limit and determine cognitive categories, there is a weaker version of the linguistic relativity hypothesis, which states that language influences cognitive processes only under certain circumstances, especially in tasks where grammatical coding is necessary (Montefinese et al., 2019). These two versions of the linguistic relativity hypothesis also apply to the issue of the influence of grammatical gender on cognitive processes (Vigliocco et al., 2005; Ramos and Roberson, 2011; Montefinese et al., 2019). Therefore the literature review presented in this paper needs to take into account studies in which grammatical gender effects were not found, as these effects may depend on circumstances such as certain types of cognitive tasks.

Prewitt-Freilino et al. (2012) distinguish three groups of languages: *genderless languages* (e.g., Finnish, Estonian, Hungarian) which do not use grammatical gender, *natural gender languages* (e.g., English, Swedish, Norwegian) in which nouns are gendered according to the biological sex of their referents, and *grammatical gender languages* (e.g., Spanish, Italian, German, as well as Polish). In case of the last language group, nouns always have a particular grammatical gender assigned to them, which influences — due to grammatical agreement — the declension and conjugation of other parts of speech. Grammatical gender languages are of great interest in the field of psycholinguistics, addressing the impact of grammatical gender on various cognitive processes, including memory, categorization, personalization, assigning properties linked to the biological sex of objects, etc. For example, grammatical gender can lead to a transfer of male and female attributes onto inanimate objects which do not have a biological sex (e.g., assigning male or female voices to artifacts depending on their grammatical gender). In general, research on grammatical gender effects focuses mainly on whether it influences various cognitive processes, especially the semantic processing of nouns. It is emphasized that interlingual differences in grammatical gender systems influence its cognitive effects. For example, numerous studies which used categorization tasks provided evidence that grammatical gender effects occur only in languages with two grammatical genders, such as Arabic (Clarke et al., 1981), Italian (Vigliocco et al., 2005), French and Spanish (Sera et al., 2002), and not in languages with more than two grammatical genders, such as German (Sera et al., 2002; Vigliocco et al., 2005). On the other hand, there is existing research in Polish – a language with more than two grammatical genders – in which grammatical gender effects were found (Ra̧czaszek-Leonardi, 2011; Haertlé, 2017).

The Polish grammatical gender system includes three main gender categories (masculine, feminine and neutral), reflected by the respective personal pronouns *ten/on, ta/ona, to/ono*. However, in plural form there also are masculine-personal and non-masculine-personal genders (*ci/oni, te/one*). Inanimate objects may have any of the grammatical genders (e.g., a tomato is *ten pomidor* (Masculine), a berry is *ta jagoda* (Feminine), and an apple is *to jabłko* (Neutral). Moreover, some beings with a defined biological sex still have neutral grammatical gender - for example *to dziecko* (child); *to szczenię* (puppy). A being with an unambiguously defined biological sex can also have a neutral grammatical gender, for example *to dziewczę* (which is a diminutive form of the word *dziewczyna* — a girl). Additionally, nouns used to describe females sometimes have a masculine grammatical gender [e.g., a young girl can be referred to as *ten podlotek* (M)] and vice versa (the Polish equivalent of ‘His majesty’ is *jego dostojność*, where *ta dostojność* is feminine). On the other hand, Polish has a lot of grammatical gender markers: personal pronouns, demonstrative pronouns, nouns, adjectives, verbs, numerals — all containing morphological information about grammatical gender, consistent with the grammatical gender of the noun. Please see Appendix 1 for a summary of word types carrying information on grammatical gender in Polish.

There are few empirical studies on the influence of grammatical gender on cognition in Polish (Ra̧czaszek-Leonardi, 2011; Haertlé, 2017). The primary goal of our research was to further investigate the effects of grammatical gender in Polish. Our research concerns the influence of grammatical gender on the categorization of words and attribution of gender-related traits to objects. We are also interested in whether this influence goes beyond verbal material – whether the grammatical gender effects will occur when objects are presented as images, rather than words. The aim of our research was also to test two groups of mechanisms by which the grammatical gender can influence selected cognitive processes, referred to as the *similarity and gender hypothesis* and the *sex and gender hypothesis* (Vigliocco et al., 2005), which will be discussed in detail further in this paper. In addition, the aim was to check whether an implicit influence of grammatical gender on the categorization process will occur in the Polish grammatical gender system.

Our research implemented three different research procedures. The first experiment used triadic similarity judgments (based on the research by Vigliocco et al., 2005), where the categorization task was to choose two words from a triad, based on the first impression about their similarity. The second experiment used a modified procedure based on the Implicit Association Test (IAT), where participants performed a task of semantic categorization of words into selected categories. The third experiment concerned the assignment of male and female voices to animals and inanimate objects, presented either as words or pictures, in order to test the link between masculine and feminine properties of referents and grammatical gender of nouns.

### Cognitive Processes Influenced by Grammatical Gender

How can the “average” language user react to the gender variation of words, especially for inanimate objects and abstract phenomena which do not have a biological sex? On the one hand, when learning a language, one could assume that the differences in grammatical gender of words provide some information about the world and are not accidental. On the other hand, one can agree that, for example, a *tomato* is not or does not become more “masculine” due to the mere fact it has a masculine grammatical gender, and *salt* does not gain feminine traits due to its feminine grammatical gender (in Polish). In linguistics it is often postulated (see Fodor, 1959) that grammatical gender of inanimate objects does not carry semantic connotations. While the grammatical gender of animate personal and some of the animate non-personal nouns has semantic foundations, assigning other nouns to particular grammatical genders may be completely arbitrary (Boutonnet et al., 2012). Indeed, in various *grammatical gender languages* particular words have the same or different grammatical genders. For example, the counterpart of the word *sun* in German is feminine (*die Sonne)*, in Spanish — masculine (*el sol*), and in Polish — neutral (*to słońce*); the word *moon* is feminine in Spanish (*la luna*) and masculine in German (*der Mond*) and Polish (*ten ksiȩżyc*). On the other hand, some researchers notice a connection between grammatical gender and semantic processing – either by researching a semantic basis for assigning grammatical gender to inanimate objects (Brugman, 1897) or by showing how grammatical gender affects cognition, like personifications of days of the week (Jakobson, 1959/1966). Contemporary empirical research in psycholinguistics provides evidence of the influence of grammatical gender on various cognitive processes, related to lexical and semantic levels of words, as well as mental representations of their referents.

There are many ways in which grammatical gender influences speakers of gendered languages. A very important effect of grammatical gender is its impact on semantic categorization. Vigliocco et al. (2005) asked participants (Italian and English speakers) to judge which two out of three presented words were the most similar in meaning. These words were nouns referring to animals and artifacts. It turned out that grammatical gender affected triadic similarity judgments in Italian speakers more than English speakers, but this effect was present only for words referring to animals and not artifacts. This study was the basis for Experiment 1 in the present paper and will be discussed in detail further on. Similarly, Cubelli et al. (2011) presented English, Italian and Spanish speakers with pairs of pictures of objects belonging to eight semantic categories (mammals, birds, vegetables, buildings, furniture, clothing, instruments, and vehicles) and asked them to decide whether the two presented objects belong to the same semantic category, measuring reaction times. Responses to gender-consistent pairs were significantly faster for Italian and Spanish speakers, but not English speakers. Moreover, articulatory suppression (participants constantly saying ‘blah, blah, blah’ during the procedure) negated the effect. A study with the use of EEG showed that there is spontaneous and unconscious access to grammatical gender when people are asked to perform a semantic evaluation of individual relationships (Boutonnet et al., 2012). As part of our research (Experiment 2) we tested implicit access to grammatical gender using a modified *Implicit Association Test* (IAT) and measuring response times as indicators of the influence of grammatical gender. Grammatical gender also affects how we imagine and personify inanimate objects and ideas. Artists and research participants personify such ideas as war and death (Segel and Boroditsky, 2011) or days of the week (Deutscher, 2010) as women or men, in accordance with their grammatical gender in a given language. For example, death (*der Tod* - masculine) tends to be represented by German artists as a male, while Spanish artists depict death (*la muerte* – feminine) as a female. Research by Segel & Boroditsky on over 750 personifications created by Italian, French, German and Spanish artists showed that depicted gender matched grammatical gender in 78% of the cases. A similar effect in a general population was reported by Sera et al. (1994) – Spanish speakers assign male or female voices to inanimate objects in accordance with their grammatical gender. This procedure was used in Experiment 3 of the present study and will be discussed in detail there.

Research also shows that grammatical gender affects the way we describe objects. Boroditsky et al. (2003) asked German and Spanish speakers to describe objects which have a different grammatical gender in these two languages. Participants described grammatically masculine objects using more stereotypically masculine qualities, and grammatically feminine objects with stereotypically feminine ones. For example, a key was described as heavy and metallic by German speakers (*der Schlüssel* – M) and as small, shiny and beautiful by Spanish speakers (*la clave* – F). Gender effects also occurred when participants were asked to assign certain features stereotypically related to one sex to nouns (e.g., masculine nouns were evaluated as “stronger” than feminine nouns; Konishi, 1993).

There are also studies on assigning stereotypically masculine/feminine qualities and grammatical gender in the Polish language. Haertlé (2017) investigated the effect of grammatical gender on the perception of objects in Polish and French speakers [following up on studies by Sera et al. (1994) and Boroditsky and Phillips (2003)]. In the first experiment, Haertlé presented a small group of native Polish and French speakers with images of objects and asked them to give these objects a male or a female voice for an upcoming cartoon. It turned out that voice gender was consistent with grammatical gender with an odds ratio of 13.74 to 1. It should be noted that there was a significant main effect for language (i.e., there were differences in the gender of attributed voices between languages, regardless of the grammatical gender of nouns). In the second experiment, the same participants were asked to assign gender-stereotypical adjectives to artifacts and natural objects varying in grammatical gender. Again, there was a significant consistency between gender-stereotypical traits and grammatical gender of the nouns in the speakers’ native language. Unfortunately the study has shortcomings – the sample size was *N* = 40 (20 Polish and 20 French speakers) and both experiments were conducted on the same participants, which could have made the aim of the study (i.e., grammatical gender as the main classification criterion) evident to them. Moreover, directly comparing two languages (i.e., reaction to Polish vs. French words which have different grammatical genders in these two languages) is never free from confounding factors (elements specific to a particular language, other than grammatical gender, which could influence participants’ answers), which is evident in the significant main effect for language in Experiment 1.

Another study in Polish and Italian (Ra̧czaszek-Leonardi, 2011) also investigated whether grammatical gender influences the way we describe objects. One group of participants was presented with objects (as pictures or nouns) and asked to use three adjectives to describe each one. Afterward, another group of participants was asked to rate whether these adjectives are suitable for masculine and feminine properties of objects. In accordance with Experiment 2 by Boroditsky et al. (2003) and Haertlé (2017), grammatical gender influenced the descriptions: adjectives used to describe grammatically feminine objects were rated as more suitable for feminine properties, and vice versa. Other studies on grammatical gender effects show that it also affects memory. Boroditsky et al. (2003) presented German and Spanish speakers with pairs of words (an object and a name). It turned out that pairs of words which shared grammatical gender were remembered more correctly than pairs of words with different genders. In another paper, it turned out that fragrances were remembered significantly better when the grammatical gender of the nouns describing them agreed with the type of fragrance (masculine or feminine; Speed and Majid, 2016).

There are some unresolved theoretical issues regarding grammatical gender effects. Primarily, it is not clear how they vary in languages with different gender systems. In various studies, grammatical gender effects seem constrained to certain languages – Vigliocco et al. (2005) found no grammatical gender effects in German, a language with three genders. The same paper showed no grammatical gender effects for inanimate objects and when objects were presented as pictures, rather than words. Moreover, there is not enough clear evidence on whether grammatical gender effects vary for different classes of items (most of the presented studies concern inanimate objects, sporadically including animals, and none of them investigates, e.g., abstract ideas which also have grammatical gender). Finally, while a large body of research shows that grammatical gender effects can be elicited using visual stimuli (pictures), few studies directly compare visual and verbal stimuli in this aspect. The present study aims to add to this body of research, while further investigating grammatical gender effects in Polish and applying a single-language framework in contrast with existing research which mostly uses comparisons across different languages. There is no clear consensus whether grammatical gender effects occur in languages with three genders – research in German shows no such effects, while the two existing studies in Polish seem to confirm the influence of grammatical gender on cognitive processes.

### Postulated Mechanisms of Grammatical Gender Effects on Cognitive Processes

It is assumed that the influence of grammatical gender on cognitive processes must be a by-product of the process of language acquisition. By learning the grammatical gender of a given noun, a person concentrates on select properties of the object consistent with its gender [e.g., when learning the word *sun*, a person from Germany (*die Sonne*) can associate it with stereotypically feminine features, while someone from Spain (*el sol*) may focus on features stereotypically related to men]. The need to refer to masculine or feminine properties may make them more important in the mental representation of the particular object, and thus more easily accessible (Boroditsky et al., 2003).

Vigliocco et al. (2005) consider two alternative (but not mutually exclusive) mechanisms by which the effects of gender could occur during language acquisition — the *similarity and gender* hypothesis and the *sex and gender* hypothesis. In the *similarity and gender* hypothesis it is assumed that words with similar syntactic and morphological properties usually have a similar meaning. Nouns with the same grammatical gender are used in the same linguistic context because in a sentence they require gender agreement with prepositions, adverbs, pronouns, etc. Grammatical gender effects are therefore a by-product of inferring semantic similarity from the linguistic context alone. As the authors point out “The basic idea is that words that have similar syntactic and morphophonological properties also tend to have similar meanings” (Vigliocco et al., 2005, p. 502). This hypothesis predicts that the effect of gender will occur both in languages with two genders (like Italian and in languages with more than two genders (German or Polish). In morphologically rich languages (i.e., ones with a large number of grammatical gender markers), similarities in the linguistic context influence cognition regardless of whether the grammatical gender of a noun reflects the biological sex of its referent. If grammatical gender effects are based on the similarity of linguistic context, one can expect grammatical gender effects to occur also for inanimate objects.

The *sex and gender* hypothesis explains the effects of grammatical gender based on the formation of relationships between the grammatical gender of nouns and the biological sex of their human or animal referents. When learning a grammatical gender language one can notice the relationship between grammatical gender and biological sex, i.e., the similarities between feminine and masculine properties and the grammatical gender of nouns, and them being shared by both linguistic properties (grammatical gender) as well as conceptual representations (biological sex). It is also assumed that there is greater semantic similarity between nouns belonging to the same gender category (Vigliocco et al., 2005). According to this hypothesis, grammatical gender effects may appear mainly for animate nouns in languages with two grammatical genders. The effects will be weaker or even absent in languages with more than two grammatical genders. This is because for languages with two genders, the discovery of the relationship between biological sex and gender should be easier, as there are no nouns with neutral or other grammatical gender, which are not diagnostic for any biological sex and/or its traits. This prediction was confirmed in research conducted in German which has three gender categories (Sera et al., 2002; Vigliocco et al., 2005), where no grammatical gender effects were found.

### Current Research

It is commonly assumed (see Vigliocco et al., 2005) that grammatical gender effects are dependent on the following properties of a language’s gender system: (1) the number of genders – languages with two genders are expected to generate stronger grammatical gender effects than languages with three or more genders; (2) the degree of correspondence between grammatical gender of nouns and biological sex of their referents (the *sex and gender* hypothesis), and (3) the extent to which parts of speech (pronouns, adjectives, numerals etc.) require gender agreement with the noun.

Grammatical gender effects, especially in a categorization task, were mainly confirmed for languages with two grammatical genders, in which it is easier to perceive a link between sex and grammatical gender. The aim of our research was to verify whether grammatical gender influences categorization processes in Polish — a language which has three genders in singular form and additional two in plural, with a large number of grammatical gender markers. We also tested grammatical gender effects on abstract nouns, in addition to inanimate and animate nouns. To our knowledge there is no research regarding grammatical gender effects on abstract nouns, at least not in Polish.

We present a series of three experiments in which we examined grammatical gender effects in Polish. In Experiment 1 (based on the research paradigm by Vigliocco et al., 2005) we used a categorization task, where participants were asked to choose two words from a triad, based on the first association about their similarity. The goal was to check whether under such conditions grammatical gender effects would be present for abstract words, names of animals and inanimate objects. In Experiment 2, based on the Implicit Association Test (IAT) paradigm, participants performed a semantic categorization task under severe time pressure. The aim was to check whether grammatical gender was implicitly accessible when performing a task that runs at the lexical and semantic level. In Study 3, we examined the attribution of male and female voices to animals and inanimate objects, presented either as words or pictures, in order to verify whether the influence of grammatical gender goes beyond the lexical and semantic levels and into visual representations, and directly comparing its effects for verbal and visual stimuli. We assumed that the obtained results will allow us to test the assumptions of the *sex and gender hypothesis* and the *similarity and gender hypothesis* in the context of the Polish language.

Based on the sex and gender hypothesis, grammatical gender effects are expected to be stronger in languages with a high consistency between the grammatical gender of nouns and the biological sex of their referents. In Polish, this consistency is restricted by the fact that there are three genders in the singular form (masculine, feminine, neutral) and two genders in the plural form (masculine-personal and non-masculine-personal), which have quite a complicated relation to their singular counterparts. Grammatical gender of nouns varies between their singular and plural forms – the non-masculine-personal plural form encompasses all singular form nouns except for masculine nouns referring to people. For example, ‘*ten pies’* (a dog) which is masculine in singular form, becomes *‘te psy’* (dogs), which is non-masculine-personal in plural, but *‘ten kowal’* (a blacksmith, masculine singular) becomes *‘ci kowale’* (blacksmiths, masculine-personal plural). The strict version of the sex and gender hypothesis would therefore predict a lack of grammatical gender effects in Polish, not unlike many published studies in German. A less constrained version of the sex and gender hypothesis would predict there to be grammatical gender effects only for nouns referring to animals and humans – with a biological sex. In the three experiments presented in this paper, we included nouns referring to animals (Experiment 1 and 3), inanimate objects (all experiments) and abstract ideas (Experiment 1).

Another factor relevant to the sex and gender/similarity and gender hypotheses is that the Polish language uses a wide variety of gender markers (see Appendix 1), making grammatical gender relevant to many parts of speech, not just to nouns. Grammatical gender needs to be considered when using verbs, numerals, adjectives, pronouns, etc. Therefore the similarity and gender hypothesis would predict that grammatical gender effects be present in all the presented experiments, using both animate and inanimate nouns. Observing grammatical gender effects for nouns referring to objects which do not have a biological sex would provide strong support to the similarity and gender hypothesis.

The presented research also aims to test two more specific research questions. Experiment 2 uses a modified implicit association test to investigate whether grammatical gender effects in Polish are present on the lexicosemantic level, and not just on the conceptual level. Experiment 3 uses a method which involves attributing masculine/feminine voices to objects in an animated movie) to test whether grammatical gender effects are present on a conceptual level. Whenever referring to lexicosemantic and conceptual levels of processing, we use the differentiation as in Vigliocco et al. (2005, p. 510): the lexicosemantic level corresponds to the meanings of words and their linguistic processing (fitting into categories, retrieving and using lexical information, etc.) without the need to create mental representations of the words’ referents. Conversely, the conceptual level is based on the mental representations of the referents and their qualities, which is non-linguistic in nature. Using language without referring to mental representations of objects is strictly lexicosemantic, while processing mental representations without using language is strictly conceptual.

The above hypotheses will be discussed in more detail in the description of each experiment. Please note that these hypotheses are, to a degree, independent – it is possible that grammatical gender reflects biological sex of animals and humans, while the similarity and gender hypothesis is true for inanimate objects. Moreover, grammatical gender effects may be present both on the lexicosemantic and conceptual levels.

## Experiment 1: Triadic Similarity Judgments

Vigliocco et al. (2005) and Kousta et al. (2008) were interested in the mechanisms through which grammatical gender can influence the evaluation of semantic similarity of words. Participants performed a categorization task in Italian and German (languages with formal gender systems) using the triadic similarity procedure. The task was to assess which of the three presented words were the most semantically similar. The triads always contained two words with the same grammatical gender, and a third one with a different gender. Words referred either to animals or artifacts. Instructions included a request to use a semantic criterion: the participants’ task was to judge which two out of three were most similar in meaning. Pairs of words chosen by the participants were classified as same-gender or different-gender. The authors tested whether there was an effect of grammatical gender on similarity classification by comparing those results with the results of an English group, which was presented with the English version of the Italian or German nouns used in the study.

In Experiment 1 by Vigliocco et al. (2005), participants were Italian and English speakers. The Italian language has two gender classes (masculine or feminine). It turned out that the effect of grammatical gender on the categorization process occurred only for animate nouns (i.e., animals). In other words, pairs of animals with the same grammatical gender in Italian were chosen significantly more often than the same pairs of animals in English; however, this effect did not occur for inanimate objects.

The experiment was then replicated in German, which is a three-gender system (masculine, feminine, and neutral) and has a less transparent correspondence between the sex of referents and the grammatical gender of nouns that refer to them. It turned out that in this language grammatical gender effects did not occur for any type of nouns. In their next experiment (conducted on Italian language users) image triads were used, instead of word triads, to illustrate the same nouns as in Experiment 1. There was no effect of grammatical gender in this case. It was concluded that grammatical gender effects do not go beyond the domain of language into the conceptual level. Results of these studies suggest that the influence of grammatical gender on the categorization process is limited to languages with two grammatical genders, it occurs in tasks requiring verbalization and is limited to animate nouns (Vigliocco et al., 2005). These results suggest that gender effects are based on a generalization of the established relationship between the gender of nouns and the sex of human referents, expanding to other gender-defined entities. Such effects occur in languages? which allow for easy mapping between the gender of nouns and human referents (e.g., Italian). In turn, the lack of effect for pictorial stimuli means that these effects appear on the lexicosemantic level, not on a conceptual level.

In Experiment 1, we used the above similarity judgment task to investigate grammatical gender effects in Polish. The study described above raises doubts about factors which may have influenced the results. The authors did not control the semantic criteria used by participants when assessing the similarity of words. The choice of two words from a triad could be influenced by factors such as an object’s function in case of tools, and taxonomic features in case of animals. For example, in the triad of Italian words (taken from the paper by Vigliocco et al., 2005): *fork* (la forchetta - F), *knife* (il coltello - M) and *hammer* (il martello - M), the criterion which seems the most apparent is the objects’ function and belonging to the “cutlery” category. That is, participants would often choose *fork* with *knife* because they match semantically, although they differ in terms of their grammatical genders. It can also happen that the same grammatical gender goes hand in hand with another criterion. For example, in the triad *zebra* (la zebra - F), *giraffe* (la giraffa - F) and *deer* or *wolf* (il cervo; il lupo - M), the place of origin (i.e., Africa) may be a clear criterion, and it may be this criterion that facilitates the choice, rather than the grammatical gender. Similarly in the triad: *tiger* (la tigre - F), *lion* (il leone - M) and *goat* (la capra - F) the predominant taxonomic criterion is being a predator, and again not grammatical gender. The criterion is particularly important, because the participants’ task was to use only the meaning of words, which could have led to a thoughtful search for the criterion to be used for categorization, while it is reported in the literature that grammatical gender effects are often subconscious or automatic (see Boutonnet et al., 2012). Research by Vigliocco et al. (2005) does not provide clear information about whether the degree of similarity between objects presented within a triad was controlled to avoid generating systematic methodological bias. Another doubt is the direct comparison between Italian and German vs. English. One could argue that language differences other than grammatical gender (word length, usage frequency, pronunciation, cultural context, etc.) could interfere with results.

In our research presented below, we took extreme care to select items which do not have obvious properties linking two words in any given triad. In addition, we modified the instruction so that it did not suggest an explicit search for a semantic criterion; instead, there was a request to choose two nouns which are associated the most with each other, based on the first impression. Moreover, we included nouns referring to abstract ideas, which should facilitate grammatical gender effects on a lexicosemantic level, as abstract ideas are difficult to imagine in a conceptual form. On the other hand, words referring to animals would be the most likely to generate grammatical gender effects on a conceptual level, as gender is a relevant trait of animals. Finally, rather than compare different languages, we conducted one-sample analyses based on a theoretical expectation of indifference and randomness, using only the Polish language.

### Materials and Methods

#### Participants

Participants were 146 native Polish speakers (96 women, 50 men) recruited from various faculties of the Jagiellonian University in Krakow. Their mean age was *M* = 32.7; *SD* = 13.34, age ranged from 18 to 69 years. All participants volunteered to take part in the study upon informed consent, and they did not receive compensation (financial or otherwise) for their participation. All experiments presented in the paper were approved by the Ethics Committee of the Jagiellonian University Institute of Applied Psychology

#### Materials and Procedure

We used a similarity judgment task such as in Vigliocco et al. (2005), which consisted of presenting participants with triads of nouns. The nouns forming the triads belonged to one of three categories: abstract nouns (e.g., structure, time), words referring to inanimate objects and words referring to animals. Each triad consisted of nouns from a single category, i.e., animals were not mixed with inanimate objects and/or abstract ideas. The complete list of stimuli is presented in Appendix 2.

Each triad consisted of two nouns of the same grammatical gender (two feminine or two masculine) and one noun of another grammatical gender. This means that each triad had either the MMF structure (two masculine words and one feminine word) or FFM structure (two feminine words and one masculine word) – in randomized order. The nouns forming the triad belonged to one of three categories: 16 abstract nouns, for example: *time* (M in Polish), *function* (F in Polish); 16 inanimate objects, for example: *key* (M), *needle* (F); 12 words referring to animals, for example: *bison* (M), *zebra* (F). As can be noted, we used fewer animals (12). This resulted from the fact that we adopted strict selection criteria. Firstly, these were animal names with a certain grammatical gender, but ones which do not indicate the biological sex of the animal, meaning that the same word refers to both the male and female of the particular animal. For example the words *lew* (lion) and *lwica* (lioness) would be avoided, as they clearly indicate the animal’s biological gender, restricting the stimuli to words such as *żyrafa* (which indicates both a male and female giraffe). Secondly, we chose animals which do not have distinctive features which could be a clear criterion for choosing two objects in a triad (e.g., if two animals came from Africa and one from the Arctic Circle, then the place of origin could easily become a criterion for categorization). Similarly, if there were two mammals and one reptile in the triad, then this could become the criterion of choice. Therefore we chose animals which all belonged to the category of exotic even-toed ungulates. The selection criterion was the similarity in appearance, behavior and the habitat of the animals. For other types of nouns, we applied a similar rule. As such, we avoided situations in which two of the items in a triad would have an obvious common feature, which could be used as a categorization criterion. Another inclusion requirement was high word usage frequency, in order to avoid a more thoughtful analysis of the meaning of the words which could take place for unknown nouns, and could cause a conscious use of grammatical gender when conducting the task (along the lines of *I don’t know the meanings of the words but they are both feminine*).

We used counterbalancing in order to ensure that different word combinations would be used. Triads were constructed using all the possible three-noun combinations (with repetitions) within each category, yielding 153 triads for abstract nouns and artifacts each, and 91 triads for animals. Out of these available triads, we created 7 sets for the abstract words and artifacts (64 triads each) and 4 sets for animals (45 triads each). In case of animals there were fewer sets of triads, because fewer words passed the strict selection process described above (6 masculine gender words and 6 feminine gender words).

Each participant evaluated one set of triads within one category of nouns (abstract, artifacts, or animals; the category of nouns was a between-group variable). The task was as follows: “*Choose from each triad the two nouns that you associate the most with each other and cross out the noun that does not fit. We ask you to make decisions quickly and rely on your first impression.*” After completing the task, participants were asked to describe the strategy they used to perform the task. The aim was to check whether participants would mention grammatical gender as the basis of their decisions, hence not meeting the assumption that grammatical gender effect is implicit.

We predicted that the influence of grammatical gender would occur primarily for nouns referring to animals. In Polish there are cases where the grammatical gender does not indicate the biological sex of the animal – but in general, the assignment of grammatical gender to animals in Polish has semantic foundations, and gender information is often consistent with the biological sex of the referent. This can lead to a generalization of the relationship between grammatical gender and the biological sex of the referents and facilitate the grammatical gender effects. Predicting the occurrence of these effects in animal names is consistent with the sex and gender hypothesis and was confirmed in other studies, where the influence of grammatical gender on the categorization process was observed only for animate nouns (Boroditsky et al., 2003; Vigliocco et al., 2005).

For artifacts, grammatical gender effects would not be consistent with the sex and gender hypothesis, however, both animals and artifacts have readily accessible mental representations, hence in both cases the participants could process the words’ referents on a conceptual level and consider their prototypical traits as masculine/feminine, therefore generating grammatical gender effects in line with the similarity and gender hypothesis.

In case of abstract nouns, it is more difficult to access mental representations, which could facilitate lexicosemantic processing. Therefore the categories of nouns used in this experiment vary in two main aspects: (1) whether the referent has a gender and (2) whether a mental representation is easily accessible.

### Results

In the post-experimental questionnaire, none of the participants indicated that they used grammatical gender as the conscious criterion for categorizing words. Words chosen by each participant were classified into same-gender and different-gender pairs. Grammatical gender consistency of individual triadic similarity judgments was converted into an average consistency measure for all judgments made by each participant, which could range from 0 (none of the judgments was consistent with grammatical gender) to 1 (all of the judgments were consistent with grammatical gender). The dependent variable was essentially the percentage of same-gender pairs selected from the triads which contained two words of one gender and one word of the other gender. In the studies by Vigliocco et al. (2005) conclusions about grammatical gender effects were drawn on the basis of a comparison of results between speakers of gendered languages (Italian, German) and English speakers (English does not have grammatical gender). Instead, using one-sample *t*-tests, we compared the obtained averages of selected same-gender pairs with the expected random chance of 1/3 (or 0.(3)). If grammatical gender does not influence categorization, and other qualities are randomized, participants should select each of the three possible pairs within a triad equally often. Results indicated that for animal words, the mean proportion of selected same-gender pairs (*M* = 0.51, *SD* = 0.15) was significantly higher than the expected 1/3; *t*(47) = 8.00; *p* < 0.001. Polish speakers chose pairs of animal words with the same grammatical gender significantly more often than what would result from random chance. The effect of grammatical gender did not occur for inanimate objects [*M* = 0.32, *SD* = 0.06; *t*(48) = −1.56; *p* = 0.12]. For abstract words, the effect was reversed; the mean proportion of same-gender pairs selected was significantly lower than random chance [*M* = 0.31, *SD* = 0.05; *t*(48) = −3.24; *p* < 0.01].

**FIGURE 1:**
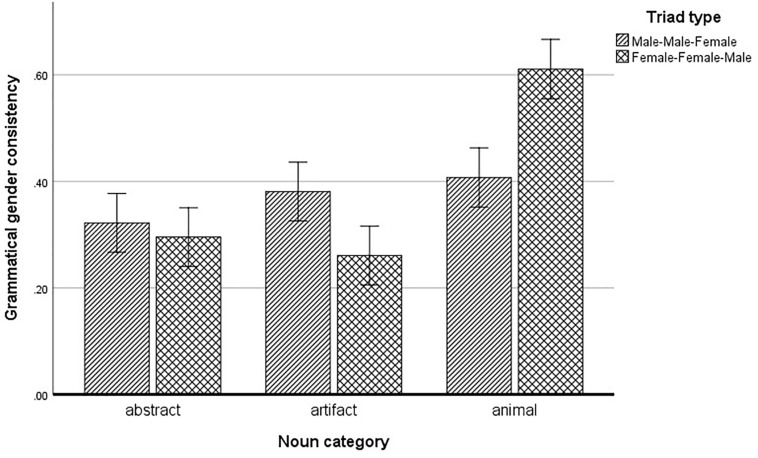
Grammatical gender consistency across triad types and noun categories.

A comparison between noun categories (abstract, artifact and animal) and triad types (MMF and FFM) using a mixed ANOVA revealed a significant main effect of noun category [*F*(2,143) = 63.44, *p* < 0.001], where artifacts and abstract nouns did not differ in categorization (*M* = 0.32 vs. *M* = 0.31, Bonferroni-adjusted *p* = 1.0), but animals (*M* = 0.51) were categorized based on grammatical gender significantly more often than artifacts and abstract nouns (both *p* < 0.001). There was no main effect of triad type [*F*(1,143) = 0.461, *p* = 0.498]. There was a significant interaction between triad type and noun category [*F*(2,143) = 11.78, *p* < 0.001]. A simple effects analysis within the interaction revealed that for artifacts, categorization was more consistent with gender in MMF triads (*M* = 0.38) than in FFM triads (*M* = 0.26; *p* < 0.014), and the opposite was true for animals (*M* = 0.40 for MMF and *M* = 0.61 for FFM triads; *p* < 0.001). Triad type had no effect on abstract ideas (*p* = 0.58). Results are presented in Figure 1.

### Discussion

Grammatical gender of nouns influenced the choice of words as similar in case of animals, but not inanimate objects, and it was reversed for abstract nouns (albeit the selection frequency differed from random chance only by about 1.4%). The result by Vigliocco et al. (2005), in which the influence of grammatical gender occurred for animals but not for artifacts was replicated in Polish. Such a result is consistent with the sex and gender hypothesis, according to which grammatical gender affects the semantic representations of gendered entities. This hypothesis also stated that in languages in which personal nouns are assigned to more than two gender categories, the influence of gender on cognitive processes is unlikely to occur and this notion was supported in a study by Vigliocco et al. (2005) in German speakers. However, in Polish, where personal nouns belong to one of three grammatical genders, the grammatical gender effect was present. We assume that this is linked to the inflection richness of this language, where nouns require gender agreement with prepositions, adverbs, pronouns and other grammatical elements of the language.

We also tested the role of grammatical gender in the case of abstract nouns, referring mainly to physical categories, such as *movement* (M), *temperature* (F), *time* (M). Their grammatical gender is arbitrary (perhaps even more than for inanimate objects), and additionally there is no easy access to the mental representations of the corresponding referents (and their traits). There was no grammatical gender effect (which can be predicted based on the sex and gender hypothesis). However, in this group of nouns a reverse effect occurred; the mean proportion of selected same-gender pairs was significantly lower than random. This may have resulted from the participants adopting a specific semantic strategy when choosing two words from the triad: the obtained result emerged from a more frequent than random selection of masculine nouns in the FFM type triads [*M* = 0.61, *SD* = 0.25, *t*(145) = 13.511, *p* < 0.001]. Numerous words with masculine gender (e.g., *condition*, *type*, *size*) referred to more general semantic categories, in which concepts defined by feminine nouns could be included. For example, the word *temperature* (temperatura – F) can be included in the broader category *condition* (stan – M). To further illustrate this point, please note that the Polish word ‘stan’ may mean *condition* as well as *state* or *class* (sociological) – it is a very broad concept. Therefore the observed effect could be a result of factors other than grammatical gender.

The fact that grammatical gender effects were present only for animals, and not for artifacts and abstract nouns, could be considered support for the sex and gender hypothesis. Even though we used nouns which do not directly state the sex of the animals, most nouns referring to animals in Polish do state whether it is a male or a female, making gender a relevant category. Moreover there is a high degree of gender consistency in the language’s inflection system. Therefore it is safe to assume that the category of gender is highly cognitively activated when thinking about animals.

Although we did not ask that participants simply refer to semantic similarity when choosing two words from a triad (the choice was to be made on the basis of the “first association”) it is possible that participants sought some semantic criteria for the selection. In the similarity judgment task, researchers have no control over the selection criteria used by participants. This applies to both Vigliocco et al. (2005; it is not known what semantic criteria were used by the participants) and the current experiment (it is impossible to control whether and to what extent the semantic factors influenced the choice of words from the triad).

Therefore we decided to check whether in Polish the effect of grammatical gender will occur for specific inanimate nouns, in a task to categorize words to unambiguously named semantic categories under strict time constraints, which facilitate implicit cognitive processing. To achieve this, we designed a task based on the *Implicit Association Test* (IAT) paradigm, where the categorization process takes place at the lexicosemantic level.

## Experiment 2: Measuring Implicit Effects of Grammatical Gender on Meaning With a Modified Implicit Association Test

In recent years, an interesting study was conducted on the hidden impact of grammatical gender on semantic processing using the category decision task. Cubelli et al. (2011) assumed that if grammatical gender and meaning are interrelated, then the assessment of whether two objects belong to the same semantic category should be influenced by the correspondence of their grammatical genders. In their study, participants were presented with pairs of pictures containing objects the names of which had the same or different grammatical genders. They were asked to decide whether these objects belong to the same semantic category. It turned out that participants (Italian and Spanish speakers) responded faster to pairs of stimuli with the same grammatical gender than ones with inconsistent gender. Compatibility of the grammatical gender of object names accelerated reactions to both semantically related pairs (positive responses) and semantically unrelated pairs (negative responses). Additionally, it turned out (Experiment 3) that this effect did not occur under conditions of articulatory suppression (when subjects were asked to repeat “blah, blah, blah” during the task). Results indicated that the grammatical gender of object names affects semantic processing in tasks requiring categorical judgments based on visual stimuli, facilitating processing of the meaning of gender-consistent noun pairs. According to the authors, the lack of gender effects under articulatory suppression (Experiment 3) means that the categorization of objects requires the processing of lexical representations and depends on the level of activation of object names. Additionally, results confirmed that grammatical gender is activated implicitly when making lexical and semantic decisions.

Boutonnet et al. (2012) reached a similar conclusion — they also used the category decision task with visual stimuli, and measured brain activity. Spanish–English bilingual participants were presented with three pictures of objects in Spanish on the screen. Their task was to assess whether the image of the third object belongs to the same or to a different semantic category as the first two, while measuring event-related brain potentials (ERPs). In half of the triads the name of the third picture had the same grammatical gender as the first two, and in the other half — the opposite one. There was no significant effect of gender consistency in the measurement of reaction times. However, it turned out that grammatical gender inconsistency modulated Left-Anterior Negativity (LAN). According to the authors, this result indicated that grammatical gender is implicitly available during categorization of objects, and this spontaneous access to grammatical gender occurs in context requiring no access to such information.

In order to check whether grammatical gender is implicitly available when performing the task of semantic categorization of words into selected categories, we used a modified *Implicit Association Test* (IAT) paradigm, first developed by Greenwald et al. (1998). In the IAT, participants classify a series of stimuli into appropriate categories, using two selected keys on a computer keyboard. The first two series and the fourth series are simple categorizations, in which the participant assigns verbal stimuli to one of two categories (e.g., good/bad and artistic/political). Series 3 and 5 include complex categorization tasks. They consist of assigning stimuli appearing on the screen to one of four categories, which are arranged in pairs in the upper corners of the screen. These are combined categories from series 1 and 2. For example, the right arrow key corresponds to both the Good and Artistic categories, and the left key to both Bad and Political. Task 5 is a reversal of Task 3: one key would correspond to Good and Political and the other to Bad and Artistic. The implicit attitudes index (the so-called IAT effect) is the difference in the response times in tasks 3 and 5; it is an indirect way of measuring the strength of the association between an object and its evaluation. For example if one has a more positive attitude toward art than politics, reaction times for Task 3 would be faster than for Task 5 (as Task 3 has a correspondence between Artistic and Good).

The goal of the present study was to verify whether the grammatical gender of nouns affects the speed and correctness of word categorization when nouns with grammatical genders (masculine and feminine) are presented along with names belonging to either a corresponding or inconsistent biological sex (men or women). Using the new method we wanted to test the assumption that gender effects appear during implicit processing at a lexicosemantic level rather than at a conceptual level – the task requires classifying words into semantic categories, which facilitates the use of lexicosemantic processing without any need or cue to recall mental representations of the referents. Based on the similarity and gender hypothesis, we predicted that RTs would be shorter and there would be fewer categorization errors for stimuli which have a consistency between their grammatical gender and the matching sex category, than for stimuli carrying an inconsistency in this aspect.

### Materials and Methods

#### Participants

One hundred and twenty eight Polish native speakers took part in the study: 30 men and 98 women aged 18–32 years (*M* = 20.97, *SD* = 2.13). Participants were students of the Faculty of Management and Social Communication of the Jagiellonian University studying various majors. Participation was voluntary and anonymous without remuneration. Informed consent was given by all participants. All experiments presented in the paper were approved by the Ethics Committee of the Jagiellonian University Institute of Applied Psychology.

#### Materials and Procedure

The modified IAT included words with masculine and feminine grammatical genders belonging to two semantic categories: clothes and trees. We excluded items of clothing which are stereotypically linked to the wearer’s sex, such as *skirt, dress, tie* – ostensibly selecting only unisex clothing names, e.g., *coat* (płaszcz - M) or *sock* (skarpeta – F). The complete list of stimuli is presented in Appendix 3. The first series was a simple categorization: there were two categories - MEN on the left and WOMEN on the right, in the upper corners of the screen. In the center of the screen, masculine or feminine names (e.g., John or Mary) were displayed. The task was to press the left CTRL key when the name belonged to the category on the left side of the screen, and to press the right CTRL key when the name belonged to the category on the right side of the screen. Participants were instructed to react quickly but correctly. In the second series, the participants classified word stimuli to simple semantic categories: CLOTHES and TREES. In one part of the experiment, the grammatical gender of clothes was masculine, and the grammatical gender of tree names was feminine, and it was reversed in the subsequent phase of the experiment (this order was counterbalanced between participants). The first two series can be considered training sessions for participants to learn the categorization task. The third series required complex categorization. Rather than a single category, pairs of categories were presented in each upper corner of the screen: MEN/CLOTHES on one side and WOMEN/TREES on the other. Stimuli belonged to all four categories: names of men, names of women, clothes and trees. Participants were to press the left CTRL button if the stimulus was a male name or a piece of clothing, and the right CTRL key if the stimulus was a female name or a tree – therefore creating correspondence between masculine – clothing, and feminine – trees. Since clothing and tree names had either a masculine or feminine grammatical gender, it was expected that RTs for grammatically masculine clothes (and grammatically feminine trees) would be shorter than for grammatically feminine clothes (and grammatically masculine trees), in case of which there would be a category inconsistency stemming from grammatical gender. Moreover, this category inconsistency might lead to more errors. The task in the fourth series was again simple categorization – the same as in the second series, but reversed: if in the second series the category CLOTHES was on the left side, and TREES on the right side, then in the fourth series TREES were on the left and CLOTHES on the right. The purpose of this series was to re-learn basic categorization after the complex task. In the fifth series, the participants performed the complex categorization task once again, however this time the categories MEN and TREES were presented together, and the categories WOMEN and CLOTHES were on the other side of the screen. This allowed an opposite measure to the one in Series 3: correspondence of grammatical gender with biological sex would cause shorter RTs for grammatically feminine clothes and masculine trees, and longer RTs for the other ones, therefore counterbalancing out factors other than gender correspondence.

In order to avoid the series order effect in the complex categorization tasks, we included versions with changed exposition order of the complex categorization tasks (series 3 and 5 were swapped in 50% of the cases).

The study was carried out in a computer lab. Participants were invited in groups of three to eight people. The room was quiet and the conditions were good for concentrating on the task.

We measured the reaction time and correctness of categorization of verbal stimuli in the series with complex categorization (series 3 and 5). The independent variable was the consistency or inconsistency of the grammatical gender of words and the gender category (MEN/WOMEN) – each stimulus was either Consistent (i.e., there was a correspondence between its grammatical gender and the gender category presented alongside its lexical category) or Inconsistent. For example if TREES/MEN and CLOTHES/WOMEN were categorized together in Series 3, a grammatically masculine tree would be Consistent (as grammatical gender corresponds to the biological sex grouped together with the category Trees), and so would a grammatically feminine piece of clothing. The same stimuli would then both be Inconsistent in Series 5, where TREES/WOMEN and CLOTHES/MEN would be grouped together.

### Results

**FIGURE 2:**
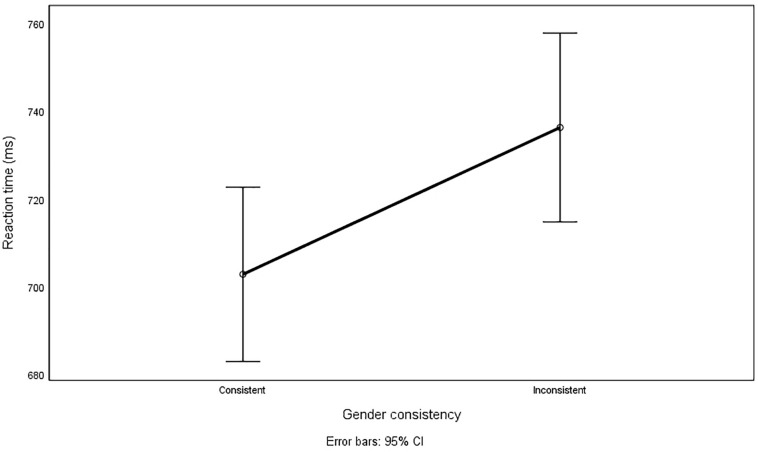
Reaction times to gender-consistent and gender-inconsistent pairs.

Analyses were conducted using a mixed ANOVA including gender consistency (Consistent vs. Inconsistent series) as a repeated measure, and the between-subjects treatments of (1) whether trees were Masculine and clothes Feminine or the other way around, and (2) the time sequence of gender consistency (Series 3 consistent, Series 5 inconsistent vs. Series 3 inconsistent, Series 5 consistent). The dependent measure was reaction time. Participants’ RTs (in milliseconds) in the gender-consistent treatment (expected marginal means *M* = 702, *SE* = 10) were significantly lower than in the gender-inconsistent treatment [*M* = 736, *SE* = 11; *F*(1,124) = 22.208, *p* < 0.001]. There was no significant interaction between Gender Consistency and item types (clothes or trees): *F*(1,124) = 0.024, *p* = 0.876. There was a significant interaction between gender consistency and time sequence – there was a larger difference between RTs in Series 3 and 5 when Series 3 was Consistent and Series 5 Inconsistent (*M* = 688, *SE* = 14 for Series 3, *M* = 783, *SE* = 15 for Series 5) than when Series 3 was Inconsistent and Series 5 – Consistent [*M* = 718, *SE* = 14 and *M* = 690, *SD* = 16, respectively; *F*(1,124) = 76.401, *p* < 0.001]. This effect can be attributed to cognitive fatigue, as participants were more tired in Series 5 than Series 3, resulting in longer RTs. The main effect of gender consistency on RTs is presented in Figure 2.

**FIGURE 3:**
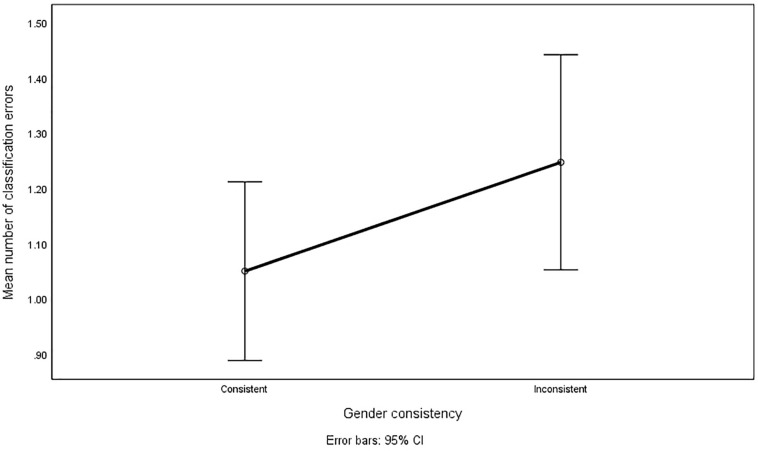
Classification errors in gender-consistent and gender-inconsistent pairs.

A separate analysis was conducted for classification errors. Again, gender consistency resulted in fewer errors (*M* = 1.052, *SE* = 0.08) than gender inconsistency [*M* = 1.25, *SE* = 0.10; *F*(1,124) = 5.441, *p* = 0.021]. The interaction with item types was non-significant [*F*(1,124) = 0.082, *p* = 0.775]. The interaction with time sequence was again significant and in the same direction as for RTs: when Series 3 was gender-consistent and Series 5 inconsistent, there were *M* = 0.803, *SE* = 0.12 errors in Series 3 and *M* = 1.540, *SE* = 0.14 errors in Series 5, and when this order was reversed there were *M* = 1.3, *SE* = 0.12 errors in the inconsistent Series 3, and *M* = 0.96, *SE* = 0.14 errors in the consistent Series 5. The main effect of gender consistency on classification errors is presented in Figure 3.

### Discussion

IAT is primarily used to study implicit attitudes by examining the process of categorizing words into affectively consistent or inconsistent categories. In our study, the consistency or inconsistency was not of an affective but rather cognitive nature.

The obtained results indicate that there was a grammatical gender effect in the task of categorizing words. Categorizations in consistent series (when masculine nouns are grouped together with masculine names, and feminine nouns with feminine names) turned out to be easier and faster than in inconsistent series (faster response times and fewer categorization errors).

The increased RTs for gender-inconsistent pairs, as compared to gender-consistent ones, suggests that grammatical gender is implicitly activated. The effect reflects a cognitive inconsistency between the grammatical genders of the stimuli and gender categories, just as the original IAT is based on affective inconsistencies between the stimuli and categories presented therein.

Despite the fact that the participants carried out the task based on semantic properties (they assigned words to categories based on their meaning), grammatical information influenced the speed and correctness of the answers. The results of this experiment support the notion that during a semantic categorization task, grammatical gender is activated implicitly. This is in line with the conclusions drawn from research by Cubelli et al. (2011) and Boutonnet et al. (2012). Semantic categorization of objects is implicitly dependent on language-specific grammatical information, such as gender, even if such information is irrelevant and not expressly elicited when performing a categorization task.

As already mentioned, Polish has a lot of grammatical gender markers, and words with the same grammatical gender require gender agreement with pronouns, adjectives, and verbs in sentences. Hence, the obtained results can be explained in line with the similarity and gender hypothesis – words which have similar syntactic and morphophonological properties also have similar meanings (Vigliocco et al., 2005).

While Experiment 2 was aimed at activating lexicosemantic processing, we planned the next experiment to use a task aimed at eliciting a conceptual level of processing, by using pictures rather than just nouns and by explicitly making gender (i.e., male and female voices) the main category to consider when making decisions.

## Experiment 3: Assigning Male and Female Voices to Animate and Inanimate Objects Presented as Pictures

One of the methods of studying the influence of grammatical gender on the categorization of objects is the task of assigning male and female voices to inanimate objects (see Sera et al., 1994, 2002). In these studies, participants were asked to assign voices to inanimate objects, which were presented in pictures with or without a label (a noun describing the object). The results for grammatical gender languages (Spanish, French, German) were compared to the results in English. It turned out that in case of Spanish and French speakers, grammatical gender had an impact on classification — the assignment of voices was consistent with the grammatical gender of objects. This effect did not occur for German speakers.

In Experiment 3, we decided to use the task of assigning voices in two conditions — presenting objects’ names or presenting images of these objects. We were interested in finding out whether grammatical gender effects will appear in Polish not only for verbal stimuli but also in non-verbal tasks (presenting images instead of nouns). The procedure used in Experiment 3 was also designed to explicitly cognitively activate the category of gender (i.e., masculine or feminine voices), which could facilitate grammatical gender effects. This is assuming that there exists a cognitive connection between the categories of grammatical gender and biological sex, which would be consistent with the sex and gender hypothesis.

In this experiment we presented the participants with a list of names or images referring to the most typical household objects and the most visually recognizable animals. The images referred to (and names were) grammatically feminine and masculine nouns. The participants’ task was to choose a masculine or feminine voice to be used by the particular object/animal if it appeared as a character in a cartoon. The experimental design was 2 (type of presentation: names vs. images) × 2 (grammatical gender of nouns: male vs. female) × 2 (animals vs. inanimate objects).

### Materials and Methods

#### Participants

One hundred people participated in this study (80 women and 20 men, aged 18–69 years; *M* = 35.2, *SD* = 14.6). The participants received one of two types of sheets: images or names. The group with images included 50 participants (42 women and 8 men) and the group with names included 50 participants (38 women and 12 men). The subjects were native Polish speakers, recruited on the street in various areas of Krakow. In the post-experimental questionnaire, none of the participants declared proficiency in foreign languages with grammatical gender. Participation was voluntary without remuneration. Informed consent was obtained from all participants. All experiments presented in the paper were approved by the Ethics Committee of the Jagiellonian University Institute of Applied Psychology.

#### Materials and Procedure

Participants were presented with nouns - names of either inanimate objects (20 words) or animals (14 words) with male or female grammatical gender – ten masculine and ten feminine inanimate objects, seven masculine and seven feminine animals. The same objects and animals were presented to another group as images. All images used in the study were icons by Freepik (available under Freepik’s free license) obtained from the website www.flaticon.com. We chose animals whose names in Polish have a specific grammatical gender (male or female), but do not indicate the biological sex of the animal (the word is the same for both sexes; see Experiment 1 for a discussion). Inanimate objects were household items, e.g., *lamp* (lampa – F) or *kettle* (czajnik – M). The complete list of nouns used in this experiment is presented in Appendix 4.

Participants were told that a therapeutic animated film for children was being created, the characters in which will be common household objects and animals. The task was to decide whether each character should speak in a woman’s or a man’s voice. Participants were asked to make quick decisions and rely on their first impression. After completing the relevant part of the study, the participants were asked to fill in a questionnaire containing a question about their strategy for making decisions about the gender of the assigned voices (in order to investigate whether grammatical gender is a consciously considered trait), as well as their proficiency in languages other than Polish (for potential cognitive interference, especially in case of images).

We assumed that performing the task of assigning male or female voices would activate the representation of objects and involve focusing on biological sex. According to the sex and gender hypothesis, grammatical gender effects should be present in case of animals. According to the similarity and gender hypothesis, there should be grammatical gender effects for animals and inanimate objects. Cubelli et al. (2011) showed that participants tend to verbalize the names of items presented in pictures. It was therefore expected that both for visual and verbal stimuli, grammatically masculine objects and animals would more often be given male voices, and vice versa – grammatically feminine objects and animals would be given female voices.

### Results

In the post-experimental survey, none of the respondents declared basing their decisions on grammatical gender. Answers (female vs. male voice) to all items were used to calculate average gender consistency ratings for M/F animals and items separately, for M/F nouns, and a total consistency index for all nouns. The consistency ratings could have any value between 0 (always inconsistent with grammatical gender) and 1 (always consistent with gender). These ratings were compared to random chance of 0.5 using one-sample *t*-tests. A strong consistency of voice with grammatical gender was present for all conditions. For masculine animals, it was *M* = 0.86, *SD* = 0.18, *t*(99) = 19.866, *p* < 0.001. For feminine animals, it was *M* = 0.78, *SD* = 0.21, *t*(99) = 13.302, *p* < 0.001. For masculine items, it was *M* = 0.81, *SD* = 0.21, *t*(99) = 14.328, *p* < 0.001. For feminine items, it was *M* = 0.79, *SD* = 0.20, *t*(99) = 13.057, *p* < 0.001. For all feminine nouns, the consistency index was *M* = 0.79, *SD* = 0.20, *t*(99) = 14.396, *p* < 0.001 and for all masculine nouns it was *M* = 0.83, *SD* = 0.16, *t*(99) = 20.015, *p* < 0.001. The total consistency index for all items was *M* = 0.81, *SD* = 0.15, *t*(99) = 20.489, *p* < 0.001.

**FIGURE 4:**
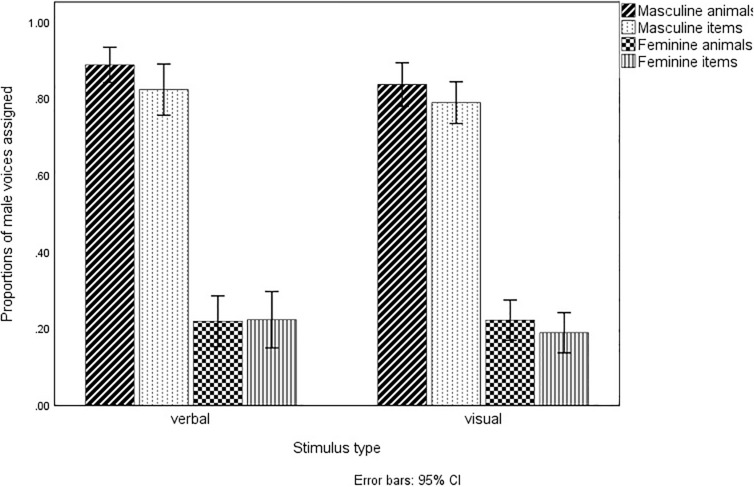
Proportions of male voices assigned by grammatical gender and stimulus type.

Another analysis was conducted to investigate differences in gender effects across treatments. It was expected that masculine nouns would be given male voices more often than grammatically feminine nouns. A mixed ANOVA with one between-subject factor (stimulus type: visual/verbal) and two within-subject factors (animal/item and feminine/masculine grammatical gender) was run. In line with previous results, it turned out that male voices were given significantly more often to grammatically masculine (*M* = 0.84, *SE* = 0.016) than feminine nouns [*M* = 0.214, *SE* = 0.02; *F*(1,98) = 38.529; *p* < 0.001]. This effect did not interact with stimulus type [*F*(1,98) = 0.210; *p* = 0.648]. Interestingly enough, male voices were given slightly more often to animals (*M* = 0.542, *SE* = 0.013) than items [*M* = 0.507, *SE* = 0.013; *F*(1,98) = 4.268; *p* = 0.041], regardless of their grammatical gender (as if animals in general are considered more ‘male’ than items). Again, this effect did not interact with stimulus type [*F*(1,98) = 0.082; *p* = 0.776]. There was also no significant interaction between grammatical gender and animal/item nouns [*F*(1,98) = 2.743; *p* = 0.101]. Fractions of male voices assigned by participants to nouns in different treatments are presented in Figure 4.

### Discussion

This experiment had two main goals. The first was to confirm the role of grammatical gender in conceptual representations in Polish and secondly, to check whether grammatical gender information transfers to non-verbal material. Images of objects and words were more often assigned a voice consistent with their grammatical gender — male voices were assigned to grammatically masculine objects and female voices to feminine objects. The influence of grammatical gender on the assignment of voice occurred both for animate (animals) and inanimate nouns (objects). Moreover, the effect was present and comparable for verbal and visual stimuli, and for each separate category.

The occurrence of such a strong effect for each category indicates the importance of gender information in conceptual representations. The effect was probably influenced by the nature of the experimental task, which consisted of assigning a female or male voice to individual objects, and thus explicitly referred to the category of biological sex, which could have cognitively activated gender information and facilitated access to it. The fact that there were no significant differences in the results between words and pictures may be associated with the tendency to quietly verbalize the names of objects when recognizing them in the pictures. As demonstrated by Cubelli et al. (2011), in case of pictures access to information about grammatical gender happens due to the activation of the name of the presented object (articulatory suppression makes the grammatical gender effect disappear). Assigning male and female voices to animals and inanimate objects according to the grammatical gender of their names shows that the grammatical gender effects in Polish may be present at the conceptual level, when gender is an explicit part of the decision task.

It is worth noting that while Experiments 1 and 2 were aimed at investigating implicit cognition under time pressure, they lacked ecological validity. In contrast, Experiment 3 uses a procedure which resembles a real-life decision of choosing what voice to give to an animated character in an upcoming movie. The procedure was akin to eliciting preferences in a survey. Results of all three experiments are therefore more generalizable to other tasks.

## General Discussion

Grammatical gender is an important syntactic phenomenon which can affect the semantic level of processing and various cognitive processes. However, research on the scope and factors of this impact delivers inconsistent results. It is assumed (see Sera et al., 2002) that this is due to, among others, interlingual differentiation of the gender systems, meaning that grammatical gender effects may vary in different languages. The purpose of our work was to investigate the extent of grammatical gender effects in Polish.

The specificity of the Polish language (5 grammatical genders which obscure the correspondence between genders of nouns and sex of their referents, but on the other hand, a large number of grammatical gender markers) prompted us to test the *similarity and gender hypothesis* and the *sex and gender hypothesis* in this language. We were also interested in whether gender effects could appear both at the lexicosemantic level and at the conceptual level.

The presented research improves upon existing studies of grammatical gender effects in Polish and in general in the following ways: In Experiment 1, we investigated the influence of grammatical gender on categorization within triads of nouns, using not only animals and inanimate objects, but also abstract nouns as stimuli. Most of existing studies use either inanimate objects or animals, and not abstract ideas. Neither inanimate objects nor abstract ideas generated the expected grammatical gender effect in our study, with the effect for abstract nouns reversed.

Experiment 2 was conducted using a method based on the IAT (Greenwald et al., 1998), which to our knowledge is the first application of this method to grammatical gender effects. The main merit of IAT is that it is based on implicit cognition. Since grammatical gender effects were present in Experiment 2, we can conclude that grammatical gender influences cognition in an implicit manner, and not just due to overt classification strategies.

Experiment 3 was largely a replication of the research by Haertlé (2017), which in turn was a replication of the study by Sera et al. (2002), however Haertlé’s research was conducted only on visual stimuli. We modified the experiment by including both visual stimuli (images) and verbal stimuli (names of the relevant objects) allowing a direct comparison between the stimuli types. Grammatical gender effects occurred for both animate impersonal nouns (animals) and inanimate nouns (objects). Moreover, the influence of grammatical gender turned out to be similar for verbal and visual stimuli.

Our research adds to the existing data showing that the grammatical gender effects are influenced by interlingual differences in the grammatical structure of the gender system. Results seem to contradict the notion that the number of grammatical genders (two vs. more than two) is crucial for determining grammatical gender effects, due to a more difficult mapping of the relations between grammatical gender and sex in languages with more than two genders. While research in German suggested that gender systems including more than two grammatical genders may not generate grammatical gender effects, these effects are present in Polish, a language with three singular and two more plural grammatical genders. This in turn may suggest that the number of grammatical genders is of lesser importance than the linguistic context at a syntactic level, and the multitude of gender markers.

The obtained results primarily support the *similarity and gender* hypothesis, which assumes that it is easier to assign similar meanings to words which have similar syntactic and morphological properties. Nouns which have the same gender are used in the same linguistic context, because the nouns’ grammatical gender requires gender agreement with numerous grammatical elements of the language. Polish is characterized by a large number of grammatical gender markers. This grammatical information can be unknowingly absorbed from the very beginning of children’s speech development.

It seems that both the *sex and gender* and the *similarity and gender* hypotheses can be used to explain grammatical gender effects in various tasks. If the experimental design explicitly activates thinking in categories of biological sex (e.g., assigning male/female voices), mechanisms consistent with the *sex and gender* hypothesis may be the cause of grammatical gender effects. In contrast, tasks which require processing on the lexicosemantic level and/or are based on implicit cognition (such as the IAT in Experiment 2), grammatical gender effects may be based on mechanisms consistent with the *similarity and gender* hypothesis. This would explain the variability of results in our research and would mean that the *sex and gender* and *similarity and gender* hypotheses are complementary.

Further research may focus on investigating the mechanisms and language aspects which cause interlingual differences in grammatical gender effects. Another interesting path for future research may use plural forms, which at least in Polish also have different grammatical genders. An interesting issue for future research involves investigating whether grammatical gender effects change along the length of the experiments, i.e., emerge over time or disappear with cognitive fatigue. Unfortunately in the presented study, the data was automatically coded item-wise, not taking into account the changes in item order in various counterbalancing sets, hence making such *post hoc* analyses impossible.

## Data Availability Statement

The datasets generated for this study are available on request to the corresponding author.

## Ethics Statement

This study was carried out in accordance with the recommendations of the Jagiellonian University Institute of Applied Psychology ethics commitee, with written informed consent from all subjects. All subjects gave written informed consent in accordance with the Declaration of Helsinki. The protocol was approved by the Jagiellonian University Institute of Applied Psychology ethics commitee.

## Author Contributions

JM: idea and conceptualization, literature survey, preparation of the method, manuscript preparation and editing, and project leader. MP: conceptualization, literature survey, statistical analyses, manuscript preparation and editing, and corresponding author. NŚ: data collection and preparation, preparation of the method, and supporting role in conceptualization.

## Conflict of Interest

The authors declare that the research was conducted in the absence of any commercial or financial relationships that could be construed as a potential conflict of interest.

## APPENDIX

**Appendix 1:** Types of words with grammatical gender in the Polish language, in singular form (with examples).

		**Masculine**	**Feminine**	**Neutral**
**Nouns**	**Referring to people**			
	Consistent with biological sex	(ten) mȩżczyzna *a man*	(ta) kobieta *a woman*	
	Inconsistent with biological sex	(ten) podlotek *a girl*	(ta) chłopina *a man (colloquially)*	(to) dziewczȩ *a girl*
	Regardless of biological sex	(ten) człowiek *a human (male/female)*	(ta) osoba *a person (male/female)*	(to) dziecko *a child (male/female)*
	**Referring to animals**			
	Consistent with biological sex	(ten) lew *a male lion*	(ta) lwica *a female lion*	
	Regardless of biological sex	(ten) hipopotam *a hippopotamus (male/female)*	(ta) żyrafa *a giraffe (male/female)*	(to) prosiȩ *a pig (male/female)*
	**Referring to inanimate objects and abstract ideas**			
	Inanimate objects	(ten) klucz *a key*	(ta) fontanna *a fountain*	(to) żelazko *an iron*
	Abstract ideas	(ten) czas *time*	(ta) forma *form*	(to) stȩżenie *concentration*
Verbs (according to grammatical gender of actor)		(on) zrobił *he did*	(ona) zrobiła *she did*	(ono) zrobiło *it did*
Numerals (according to grammatical gender of relevant verb)		pierwszy *first (masculine)*	pierwsza *first (feminine)*	pierwsze *first (neutral)*
Pronouns (according to grammatical gender of relevant verb)		on *(he)* mój *(mine, masculine)*	ona *(she)* moja *(mine, feminine)*	ono *(it)* moje *(mine, neutral)*
Adjectives (according to grammatical gender of relevant verb)		ładny *pretty (masculine)*	ładna *pretty (feminine)*	ładne *pretty (neutral)*

**Appendix 2:** Items used in Experiment 1.

**Abstract nouns**		**Inanimate obj.**		**Animals**	
**Polish (English)**	**GG**	**Polish (English)**	**GG**	**Polish (English)**	**GG**
temperatura (temperature)	F	żarówka (lightbulb)	F	zebra (zebra)	F
siła (strength)	F	doniczka (flowerpot)	F	żyrafa (giraffe)	F
struktura (structure)	F	klawiatura (keyboard)	F	antylopa	F
waga (weight)	F	cegła (brick)	F	(antelope)	F
pozycja (position)	F	igła (needle)	F	lama (llama)	F
funkcja (function)	F	fontanna (fountain)	F	gazela (gazelle)	M
barwa (color)	F	czekolada (chocolate)	F	alpaka (alpaca)	M
forma (form)	F	obroża (collar)	F	wielbła̧d (camel)	M
czas (time)	M	klucz (key)	M	bawół (buffalo)	M
ruch (motion)	M	parasol (umbrella)	M	bizon (bison)	M
stan (condition)	M	sznur (rope)	M	żubr (aurochs)	M
dźwiȩk (sound)	M	plecak (backpack)	M	łoś (moose)	M
rozmiar (size)	M	czajnik (kettle)	M	renifer (reindeer)	
rodzaj (type)	M	zegar (clock)	M		
smak (taste)	M	pocisk (bullet)	M		
zapach (smell)	M	bilet (ticket)	M		

*M, masculine; F, feminine.*

**Appendix 3:** Items used in Experiment 2.

		**Part 1**		**Part 2**	
**Part 1 Names**	**Sex**	**clothes (M) and trees (F)**	**GG**	**clothes (F) and trees (M)**	**GG**
		**Polish (English)**		**Polish (English)**	
Katarzyna	F	brzoza (birch)	F	bluza (sweatshirt)	F
Magdalena	F	kapelusz (hat)	M	buk (beech)	M
Tomasz	M	płaszcz (coat)	M	grab (hornbeam)	M
Jan	M	sosna (pine)	F	czapka (cap)	F
Mateusz	M	wierzba (willow)	F	kurtka (jacket)	F
Anna	F	szalik (scarf)	M	modrzew (larch)	M
Ewa	F	akacja (acacia)	F	klon (maple)	M
Michał	M	jodła (fir)	F	kasztan (chestnut)	M
łukasz	M	podkoszulek (undershirt)	M	rȩkawiczka (glove)	F
Kinga	F	sweter (sweater)	M	kamizelka (vest)	F
Jacek	M	topola (poplar)	F	da̧b (oak)	M
Natalia	F	lipa (linden)	F	skarpetka (sock)	F
Karolina	F	jarzȩbina (rowan)	F	jesion (ash)	M
Agnieszka	F	golf (turtleneck)	M	świerk (spruce)	M
Stanisław	M	dres (tracksuit)	M	koszula (shirt)	F
Adam	M	szlafrok (bathrobe)	M	piżama (pajamas)	F

**Appendix 4:** Items used in Experiment 3.

**Inanimate objects**		**Animals**	
**Polish (English)**	**GG**	**Polish (English)**	**GG**
miotła (broom)	F	żaba (frog)	F
wanna (bathtub)	F	żyrafa (giraffe)	F
kanapa (sofa)	F	mysz (mouse)	F
pralka (washing machine)	F	wiewiórka (squirrel)	F
szklanka (cup)	F	małpa (monkey)	F
suszarka (dryer)	F	ośmiornica (octopus)	F
lampa (lamp)	F	sowa (owl)	F
lodówka (fridge)	F	hipopotam (hippopotamus)	M
szafa (wardobe)	F	żółw (turtle)	M
żarówka (lightbulb)	F	wa̧ż (snake)	M
fotel (chair)	M	jeż (hedgehog)	M
klucz (key)	M	goryl (gorilla)	M
wieszak (coat hanger)	M	nietoperz (bat)	M
zegar (clock)	M	kangur (kangaroo)	M
garnek (pot)	M		
nóż (knife)	M		
czajnik (kettle)	M		
odkurzacz (vacuum cleaner)	M		
obraz (picture)	M		
stół (table)	M		
